# The Seaford Mock-Marriage Case

**Published:** 1912-08-24

**Authors:** Willoughby Bullock

**Affiliations:** Secretary and House Governor.


					The Seaford Mock-Marriage Case.
To the Editor of The Hospital.
Sir,?As the proceedings over the " Seaford Mock-
Marriage" case have received so much publicity in the
press, and in various accounts it is stated that the
.-affair originated at "The Seaford Convalescent Home,"
may I ask you to be good enough to publish the fact that
'the Seaside Convalescent Hospital, of which H.M. the
King is Patron and Sir William Bull, M.P., Chairman,
is in no way concerned ?
We are the only convalescent institution in Seaford,
;and for some reason or other are far better known to the
general public and to a number of our supporters as the
.Seaford Convalescent Home than by our correct designa-
tion, and in this case our identity appears to have been
?confused with another somewhat similar institution in
the vicinity where one party was a patient and the other
a member of the staff.
As we are entirely dependent on public subscriptions
for our existence you will, I am sure, readily see
the necessity for the publication of this explanation.?
I remain, yours very truly,
WlLLOUGHBY BULLOCK,
Secretary and House Governor.
August 20.

				

